# A DNA barcode library for mangrove gastropods and crabs of Hong Kong and the Greater Bay Area reveals an unexpected faunal diversity associated with the intertidal forests of Southern China

**DOI:** 10.1186/s12862-021-01914-6

**Published:** 2021-09-23

**Authors:** Henrique Bravo, Christine L. Y. Cheng, Alessio Iannucci, Chiara Natali, Aline Quadros, Martin Rhodes, Matthew M. L. Yip, Stefano Cannicci, Sara Fratini

**Affiliations:** 1 The Swire Institute of Marine Science and Division for Ecology and Biodiversity, School of Biological Sciences, The University of Hong Kong, Pokfulam Road, Hong Kong, Hong Kong S.A.R. People’s Republic of China; 2 Groningen Institute for Evolutionary Life Sciences, University of Groningen, 9700 CC Groningen, The Netherlands; 3 Department of Biology, University of Florence, 50019 Sesto Fiorentino, Italy

**Keywords:** Biodiversity monitoring, Mangrove faunal diversity, DNA barcoding, MOTUs, Gastropoda, Brachyura

## Abstract

**Background:**

Mangroves are tropical and subtropical intertidal forests colonising sheltered coasts across the world. They host a unique faunal community, dominated by brachyuran crabs and gastropods. These invertebrates strongly contribute to the functionality of the entire forest. The reliable assessment of mangrove faunal diversity is, thus, a crucial step for efficient management and conservation plans, but it is hindered by difficulties in species identification. Here we provide a verified DNA barcode library for brachyuran crabs and gastropods inhabiting the mangroves of the Greater Bay Area, Southern China. In particular, we collected and morphologically identified 1100 specimens of mangrove associated brachyuran crabs and gastropods. The partial sequences of the mtDNA cytochrome oxidase subunit I gene were obtained from 275 specimens. Barcode sequences were then used to delineate Molecular Operational Taxonomic Units (MOTUs), employing three different delimitation methods: the automatic barcode gap discovery (ABGD) method, the general mixed Yule coalescent (GMYC) model and a Bayesian implementation of the Poisson tree processes (bPTP) model.

**Results:**

By integrating DNA barcodes with morphology, we identified 44 gastropod species and 58 brachyuran species associated with Hong Kong mangroves, with five and seven new records, for gastropods and crabs, respectively, for the Greater Bay Area. The delineation of MOTUs based on barcode sequences revealed a strong congruence between morphological and molecular identification for both taxa, showing the high reliability of the barcode library.

**Conclusions:**

This study provides the first reference barcode library for mangrove-associated macrobenthic fauna in the Greater Bay Area and represents a reliable tool to management and conservation plans. Our molecular analyses resolved long lasting taxonomic misidentifications and inconsistencies and updated the knowledge on the geographical distribution of Asian mangrove associated fauna, ultimately highlighting a level of biodiversity higher than previously thought for Southern China.

**Supplementary Information:**

The online version contains supplementary material available at 10.1186/s12862-021-01914-6.

## Background

Despite accounting for only 0.7% of the total tropical forests of the world [1], mangroves play a critical role in the protection of coastal areas, act as nurseries for many species, sequestrate carbon, recycle nutrients, and provide benefits to people through the direct exploitation of their resources [2–5]. Such ecosystem services are provided by a unique biome represented by a floral community of terrestrial origin and the specialised marine and intertidal macroinvertebrate assemblages thriving in these forests [2, 6–8].

Significant decreases of either mangrove forest area cover [9] or of their functionality and service provision [10] have been witnessed over the last decades. Despite the current rates of mangrove deforestation having slowed down in the last decade, the loss and degradation have not been reversed yet [11]. Alongside urban and coastal development, aquaculture, rice plantations, palm oil plantations and climatic changes are still the main factors responsible for the rapid deforestation of mangroves [12–15]. Conservation efforts and good restoration practices are therefore paramount to preserve functional and viable mangrove patches, and they need to be put in place rapidly [11].

Mangrove-associated macrobenthos communities are mainly represented by decapod crustaceans and molluscs, whose diversity peaks in the Indo West Pacific area, i.e., the centre of origin of mangrove forests in the Late Cretaceous [16]. Many of these species are exclusive inhabitants of these intertidal forests and show a high degree of adaptation to the mangrove environment. Among brachyuran crabs, the families Sesarmidae, Ocypodidae, Dotillidae, Macrophthalmidae and Varunidae are represented the most in terms of species diversity, relative abundance and adaptations [6, 17–19]. The dominant molluscan taxa are represented by gastropods belonging to Littorinidae, Potamididae, Onchidiidae and Ellobiidae families [16, 20, 21], which also show unique adaptations to intertidal life.

Although it is now well understood that a rich and functionally redundant fauna is the key to ensure full mangrove functionality [7], taxonomic surveys and species identification have proved to be challenging for mangrove associated macrofauna, mainly due to the cryptic behaviour of many species, inaccessibility to some areas, and the presence of a high number of morphologically very similar, and phylogenetically related, species. Recent studies have circumvented the identification issues by using DNA barcodes to identify the presence of species in mangrove forests [20, 22–24]. DNA barcoding for metazoans focuses traditionally on the use of the mitochondrial gene cytochrome *c* oxidase subunit 1 (COxI) to identify diversity amongst sequences of different individuals [25]. This method has proven reliable to distinguish species of different taxa, including crustaceans and molluscs [26]. Access to barcodes assigned to species from unverified sources, however, may still make it difficult to accurately identify specimens without expert morphological knowledge. This uncertainty can lead to misidentification of specimens [27], which can result in a wrong evaluation of the taxonomic diversity and functional redundancy of a mangrove forest and, ultimately, of its vulnerability. A complete DNA barcoding library based on verified specimen identifications would facilitate community assessments and allow the resolution of taxonomic uncertainties. Since a reliable assessment of faunal communities is a crucial need for efficient mangrove management and conservation efforts, a verified DNA library is an important tool to establish the health, functionality and vulnerability of these threatened forests.

Hong Kong, and the Greater Bay Area, are characterised by one of the highest human densities on the planet, resulting in smaller and fewer pristine coasts and natural intertidal areas. The geographical location of the Pearl River delta in the northern part of the Tropical Oriental Region and close to the Temperate Palaearctic Japonica Region along the Western Indo-Pacific coast [28] supports a unique assemblage of marine species, characterised by high levels of biodiversity [29]. The mangroves in this area are equally diverse because the Hong Kong S.A.R. territory is located on the coast of Guangdong, the richest Chinese Province in terms of mangrove area that comprises 2.10% of the total mangrove area of China [10]. These mangrove forests are located on the outer margins of a large river delta and are characterised by different, and sometime contrasting, ecological factors [30] that support over 100 different species of molluscs and crustaceans [31, 32]. Among these, some notable endemisms are recorded, such as the arboreal micro-mangrove crab *Haberma tingkok* [33]. However, no updated or complete lists of mangrove-associated macroinvertebrates are available.

An in-depth knowledge of the composition of faunal communities across the Greater Bay Area, including cryptic and rare species, is needed to understand which mangrove forests are functionally viable and which are more vulnerable, and this will boost the ability of stakeholders, policy makers and environmental managers to design and deploy effective conservation strategies. The aim of this study was to create a reliable tool to assist researchers, conservationists and managers in assessing mangrove-associated crab and mollusc assemblages in Hong Kong and the whole Greater Bay Area. The objectives were to (1) create a complete DNA barcode library for all known brachyuran crabs and gastropods inhabiting mangrove forests in the Pearl River Delta, and (2) to resolve long lasting species misidentifications and discrepancies between morphological and genetic identifications to achieve a reliable and updated list of mangrove-associated fauna. We argue that this library, and the associated species list, not only represent a useful inventory of the biodiversity of mangrove-associated crabs and molluscs in Hong Kong and the whole Greater Bay Area, but also are a key tool for mangrove biodiversity monitoring programs in China and the whole of East Asia.

## Results

Based on the morphological assignment, we identified 44 gastropod and 58 crab species (Additional file 1: Table S1). Seven species of crabs and five species of gastropods are new records for the Greater Bay Area.

Barcode sequences were recovered from 128 gastropods and 147 crabs from the 285 samples selected for analysis, a success rate of more than 96%. Following the morphological assignment, the sequences corresponded to 43 gastropod species in 13 families and 25 genera (Tables 1 and 2). Only one gastropod species, *Sermyla riqueti*, belonging the family Thiaridae was not included in the COxI dataset because this species is rare in Hong Kong mangroves and we only had a single small specimen available for genetic analysis, from which we did not recover a reliable barcode sequence.

**Table 1:** List of the 43 gastropod species sequenced at the barcode COxI region

Family	Species	No. of barcoded specimens
Assimineidae	*Optediceros breviculum*	3
Batillariidae	*Batillaria attramentaria*	3
	*Batillaria cumingii*	1
	*Batillaria zonalis*	2
Cerithiidae	*Cerithium coralium*	2
	*Clypeomorus* sp.	3
Ellobiidae	*Cassidula aurisfelis*	2
	*Cassidula plecotrematoides*	1
	*Ellobium chinense*	1
	*Ellobium* sp.	3
	*Laemodonta punctatostriata*	2
	*Phythia* sp.	1
Haminoeidae	*Bakawan puti*	1
Littorinidae	*Littoraria ardouiniana*	3
	*Littoraria articulata*	3
	*Littoraria melanostoma*	1
	*Littoraria sinensis*	1
Nassariidae	*Nassarius sinarum*	1
	*Reticunassa festiva*	1
Neritidae	*Clithon oualaniense*	4
	*Clithon sowerbianum*	5
	*Neripteron cornucopia*	1
	*Neripteron violaceum*	2
	*Nerita balteata*	1
	*Nerita planospira*	1
	*Nerita yoldii*	6
Onchidiidae	*Laspionchis boucheti*	9
	*Onchidium stuxbergi*	1
	*Paromoionchis tumidus*	2
	*Platevindex mortoni*	1
	*Platevindex s*p.	3
	*Wallaconchis graniferus*	2
Planaxidae	*Planaxix sulcatus*	2
Potamididae	*Cerithidea tonkiniana*	1
	*Cerithidea moerchii*	2
	*Pirenella alata*	15
	*Pirenella asiatica*	2
	*Pirenella incisa*	10
	*Pirenella nanhaiensis*	4
	*Pirenella pupiformis*	4
	*Terebralia sulcata*	11
Trochidae	*Monodonta labio*	2
Turbinidae	*Lunella granulata*	2

**Table 2:** List of the 58 brachyuran crab species sequenced at the barcode COxI region

Family	Species	No. of barcoded specimens
Dotillidae	*Dotilla wichmanni*	1
	*Ilyoplax formosensis*	3
	*Scopimera intermedia*	3
	*Tmethypocoelis ceratophora*	3
Grapsidae	*Metopograpsus frontalis*	4
	*Metopograpsus quadridentatus*	2
Leucosiidae	*Philyra malefactrix*	2
Macrophthalmidae	*Ilyograpsus paludicola*	1
	*Macrophthalmus definitus*	2
	*Macrophthalmus convexus*	3
	*Macrophthalmus erato*	2
	*Macrophthalmus pacificus*	3
	*Macrophthalmus tomentosus*	2
	*Venitus latreillei*	1
Myctiridae	*Myctiris brevidactylus*	3
Ocypodidae	*Austruca lactea*	3
	*Gelasimus borealis*	5
	*Ocypode ceratophthalmus*	2
	*Ocypode sinensis*	1
	*Paraleptuca splendida*	2
	*Tubuca acuta*	3
	*Tubuca arcuata*	3
	*Tubuca paradussumieri*	2
Oziidae	*Epixanthus frontalis*	1
Pilumnidae	*Heteropilumnus sasekumari*	1
Portunidae	*Scylla serrata*	1
	*Scylla paramamosain*	1
	*Thranita crenata*	2
Sesarmidae	*Chiromantes haematocheir*	7
	*Clistocoeloma cf. merguiense*	4
	*Clistocoeloma villosum*	2
	*Episesarma versicolor*	5
	*Fasciarma fasciatum*	2
	*Haberma tingkok*	2
	*Nanosesarma minutum*	3
	*Nanosesarma pontianacense*	2
	*Neosarmatium indicum*	5
	*Orisarma dehaani*	4
	*Orisarma neglectum*	1
	*Orisarma patshuni*	2
	*Orisarma intermedium*	3
	*Parasesarma affine*	3
	*Parasesarma bidens*	5
	*Parasesarma pictum*	3
	*Parasesarma tripectinis*	2
	*Parasesarma ungulatum*	3
	*Sarmatium germaini*	3
	*Sarmatium striaticarpus*	2
	*Sinosesarma tangi*	1
Varunidae	*Chasmagnathus convexus*	3
	*Gaetice depressus*	2
	*Helicana doerjesi*	1
	*Helice latimera*	2
	*Hemigrapsus penicillatus*	2
	*Metaplax longipes*	2
	*Metaplax tredecim*	7
	*Varuna yui*	2
Xanthidae	*Leptodius affinis*	3

The crab dataset was based on 150 COxI sequences (147 original sequences and 3 sequences downloaded from Genbank) and included all the crab species we recorded in Hong Kong mangroves (58 species in 12 families and 38 genera; Tables 1 and 2).

The gastropod DNA barcode dataset included 15 species represented by a single specimen, and 28 species represented by an average of four specimens (range 2–15; Table 1). The crab DNA barcode dataset included 11 species represented by a single specimen, and 47 species represented by an average of three samples (range 2–7; Table 2).

All sequences did not present stop-coding regions and their length varied between 508 and 658 bp (i.e., the full-length barcode region) for gastropods and between 527 and 658 bp for crabs. We obtained the full-length barcode region for 58.6% of the gastropod sequences and 64% of the crab sequences. The gastropod dataset included 354 (53.8%) variable sites, and the crab dataset included 298 (44.5%) variable sites. The average nucleotide frequencies for gastropods were 22.4% adenine (A), 18.8% cytosine (C), 20.6% guanine (G) and 38.2% thymine (T); while the average nucleotide frequencies for crabs were 28.6% (A), 18.8% (C), 16.9% (G), and 35.7% (T).

A three-gap deletion in position 106–108 bp of the gene (corresponding to amino acid position 36 of the protein) was recorded in 28 sequences retrieved from the Eupulmonata gastropod species belonging to the families Ellobiidae (6 species) and Onchidiidae (6 species) (Additional file 1: Table S1).

The K2P genetic distances within species, genera and families for crabs and gastropods are summarised in Table 3. We observed a hierarchical increase in the mean K2P genetic divergence with increasing taxonomic levels from intra-specific to intra-generic and intra-family (Table 3). We found a minimum average K2P congeneric divergence of 10.18% for gastropods and 3.71% for crabs, excluding the pairwise comparison between *Orisarma neglectum* and *O. dehaani* (see MOTUs assignment results). These values were found to be about 16 and 11 times higher than the average gastropod and crab intra-specific divergence (Table 3) indicating the presence of distinct specific boundaries among the studied species.

**Table 3:** Summary of genetic divergences (K2P percent) at various taxonomic levels

	Comparisons within	Distance		
		Mean ± SE	Minimum (%)	Maximum (%)
Gastropods	Species	0.63% ± 0.25%	0.00	1.84
	Genus	18.79% ± 2.38%	10.18	28.37
	Family	25.51% ± 2.91%	20.05	33.50
Brachyuran crabs	Species	0.32% ± 0.15%	0.00	1.57
	Genus	12.28% ± 1.68%	6.50	22.37
	Family	20.07% ± 2.42%	11.42	24.71

The delineation of MOTUs revealed a strong congruence between morphological and molecular identification for both gastropods and crabs, confirming the high reliability of the barcode library. For gastropods, the ABGD analysis identified exactly 43 MOTUs (prior maximal distance of 0.0599) out of 43 morphologically identified species sequenced, showing that the sampled species can be identified unambiguously by DNA barcoding. The GMYC analysis also produced results in line with the morphological identification, identifying 44 distinct entities with a confidence interval ranging from 43 to 46. Only *Pirenella pupiformis* specimens were split in two different entities. Indeed, *P. pupiformis*, together with *P. asiatica*, were the species with the highest K2P intra-specific genetic distances (1.61% ± 0.37% and 1.84% ± 0.58%). The bPTP analyses split the dataset into a slightly higher number of clusters, returning an estimation of a number of species between 43 and 53, with a mean value of 46.29.

Out of 58 morphologically identified crab species, 56 were well delimited through the ABGD tool at a prior maximal distance of 0.0359. Only two species, *Orisarma dehaani* and *O. neglectum*, were lumped together in a single MOTU, but these are easily distinguished by morphological characters and colour [34]. Similarly, GMYC analysis yielded 59 entities with a confidence interval ranging from 57 to 63. Results were consistent with the ABGD analysis, i.e., all species were delimited except the pair represented by *O. dehaani* and *O. neglectum*: their pairwise K2P inter-specific genetic distance was 0.68% ± 0.25%, a value basically identical to intra-specific K2P genetic distance recorded for *O. dehaani* (0.69% ± 0.27%). Conversely, *Parasesarma pictum* and *Tubuca arcuata* specimens (whose intra-specific K2P genetic distances were equal to 1.57% ± 0.43 and 1.47% ± 0.40, respectively) were split in two different entities. The bPTP analyses separated the samples into more clusters, giving an estimation of a number of species between 63 and 91 with a mean value of 73.83.

## Discussion

### A reliable barcode library for the mangrove macrobenthos of the Greater Bay Area

The correct identification of species occurring in a given ecosystem is the first critical step needed to evaluate species richness and abundance and, ultimately, to plan effective management and conservation strategies. This study is the first attempt to combine an intensive sampling and DNA barcoding campaign on the macrobenthic fauna associated with Hong Kong and Greater Bay Area mangroves. Our results, based on the collection of about 1100 specimens and the molecular characterisation of about 280 specimens, identified 44 species of gastropods and 58 species of crabs associated with the largest and most pristine mangroves of the Greater Bay Area [10], and provided a barcode library that included all the species (except for one gastropod species, *Sermyla riqueti* that is rarely associated with Hong Kong mangroves and more abundant on mud flats).

The strength of the library was tested by three different species-delimitation methods. Both the distance-based method (ABGD) and the phylogeny-based methods (GMYC and bPTP) yielded species delimitation results highly concordant with morphological species assignment, pointing towards a high reliability of the library. ABGD and GMYC revealed an almost univocal correspondence between the morphological and molecular species identification. The only exception amongst crabs was the assignment of *Orisarma dehaani* and *O. neglectum* to the same MOTU, indicating that they cannot be distinguished based on their COxI sequence alone. These species are easily distinguished by various morphological traits of their carapace, by their distinctive colour patterns, and by their habitat preferences as suggested by Schubart and Ng [34]. MtDNA can fail to distinguish either recently separated sister species or hybrids, due to its evolutionary properties such as maternal inheritance, low effective population size and low mutation rate [35]. This has also been recorded in congeneric brachyuran species, for example in mangrove sesarmid crabs [36].

On the other hand, the bPTP methods produced a slightly higher number of MOTUs both for the crab and the gastropod datasets. This could be ascribed to several reasons since the performance of phylogeny-based methods is sensitive to higher substitution rates, number of species included, uneven sampling, number of singletons in the input trees and unresolved nodes [37–39]. For this, the bPTP method, despite proven to be appropriate for some studies, was already found to over-split datasets in other cases [40, 41].

### New records for Hong Kong mangrove associated fauna

This study reports several new species records for the Hong Kong SAR territory and the whole Greater Bay Area. Five out of the 44 gastropod species are new to the study area: *Nerita planospira*, *Bakawan puti* and three Onchidiidae species, *Onchidium stuxbergi*, *Laspionchis boucheti, Wallaconchis graniferus*. The *N. planospira* specimen was collected on a branch in a mangrove patch at the eastern coast and matched the descriptions in Eichhorst [42]. Hong Kong is well within the known distributional limit of this species [42]. The Haminoeidae *B. puti* was collected on Lantau Island and it was previously found only on two islands of the Philippines [43]. The taxonomy of the family Onchidiidae has long been unclear and the discovery of three new records of such mangrove-dwelling slugs in the forests of Hong Kong does not come as a surprise. The Greater Bay Area is located within the northern limit of the distributional range of *O. stuxbergi* [44] but it represents the new northernmost known limit for both *W. graniferus* [45] and *L. boucheti* [46], and the first record of the latter species from China. All these new records for gastropods are included in the barcode library.

Seven of the 58 crab species are new records to Hong Kong, namely *Ilyograpsus paludicola*, *Helicana doerjesi*, *Heteropilumnus sasekumari*, *Clistocoeloma villosum*, *Nanosesarma pontianacense*, *Sarmatium striaticarpus* and *Orisarma neglectum*. COxI gene sequences for all these species are included in the library produced in this study. The few *I. paludicola* specimens were only found on the western coast and were identified by the four teeth along the lateral margin of the carapace [47]. A single specimen of *H. doerjesi* was collected in a small mangrove artificially planted in Starfish Bay, within Tolo Harbour, a sandy area where mangroves were never recorded before [28]. It is highly likely that this rare species is only loosely associated with mangroves in Hong Kong. A population of *H. sasekumari* was found in a sparse mangrove patch on the eastern coast. *H. sasekumari* is one of the few pilumnids inhabiting mangroves and our record represents the new northernmost limit of its distribution [48]. The recorded population of *C. villosum* matches the descriptions of Yuhara et al. [49] and Komai et al. [50] and was found in two stony mangrove patches on the north eastern coast. This species has been previously recorded through a wide Indo-Pacific range [49]. A single female of *N. pontianacense* was found in the western coast mangroves, while large populations are found on artificial oyster reefs near the forest, suggesting that this small species is not a strict mangrove dweller. This is another predominantly tropical species that ranges from Borneo to Hainan in China [51] and Hong Kong is its northernmost limit. A female and a male of *S. straticarpus* were collected from two mangrove patches of the eastern coast of Hong Kong and were unequivocally identified using the descriptions in Davie [52]. The species has been recorded in Singapore, Malaysia, southern Okinawa (Japan) and the Philippines [52].

The only specimen of *O. neglectum* we found was a male from Mai Po, the largest mangrove forest within the Greater Bay Area, that was identified using the descriptions in Schubart and Ng [34]. Before this record, this species has only been recorded north of Fujian, China [34] and Hong Kong represents its southernmost distributional limit.

We also confirmed the presence of two brachyuran species previously mentioned in grey literature only, *Philyra malefactrix* and *Episesarma versicolor*. *P. malefactrix* is a mud dwelling small species, rare in Hong Kong, and it was found at the same locality as the previously known specimens, on the western coast. *E. versicolor* represents the largest sesarmid of Hong Kong mangroves and it is common and widespread on both the East and West coasts. For both these species, Hong Kong represents the northernmost limit of their distribution.

It is worth noting that we did not record any non-native species of either brachyuran crabs or gastropods during such an extensive survey. Occurrence of non-native species have long been known for Hong Kong and have caused negative impacts on native ecosystems, crop production, and public health, such as the case of the red fire ant, *Solenopsis invicta*, [53]. Introduced plant species, namely the water hyacinth (*Eichhornia crassipes*) and two species of apple mangroves (*Sonneratia* spp.) are now commonly found in both freshwater and brackish wetlands colonised by local species of mangrove plants [54, 55]. In terms of fauna, the invasive apple snail, *Pomacea canaliculata*, is abundant in Hong Kong freshwater wetlands and can impact these habitats by outcompeting native snails [56] and predating on amphibian eggs [57]. In this local context, the lack of non-native invertebrate species in mangrove forests confirms the current hypothesis postulating that mangroves are a uniquely harsh environment where only well adapted and specialised fauna can deal with the ecological and physiological challenges posed by these forests [7, 19, 58].

Although we did not record any non-native species, it should be noted that Hong Kong represents the northernmost distribution limit of most of the newly recorded species of both brachyuran crabs and gastropods found during this survey. We cannot exclude the possibility that these new records of mainly tropical species could be the results of recent range expansion events related to increased mean sea surface temperatures, as hypothesised for a recent northward range expansion of a tropical sand-bubbler crab, *Scopimera curtelsona*, from Hainan to Hong Kong [59].

### Solving of misidentifications and taxonomic inconsistencies

The barcode library presented in this study allowed us to resolve some long-lasting misidentifications and taxonomic inconsistencies in the gastropod and crab families associated with the mangroves of the Greater Bay Area.

#### Family Batillariidae (Gastropoda)

Our results reveal strong inconsistencies for the previous records of the genus *Batillaria* in Hong Kong. Our specimens that morphologically match the descriptions of *B. zonalis* and *B. multiformis* [60, 61] were attributed to *B. cumingii* [62] and *B. zonalis* [21, 62], respectively, by DNA barcodes. Similar inconsistency between morphology and COxI gene was reported in [63], where some shells that morphologically resembled *B. flectosiphonata* were shown to be *B. cumingi* and *B. multiformis*.

#### Family Ellobiidae (Gastropoda)

*Ellobium aurijudae* was previously recorded in Hong Kong [28, 32] but, although the shell of our specimens corresponds to the available descriptions for *E. aurisjudae* [64], their DNA barcodes are different from the ones from *E. aurisjudae* collected in Southeast Asia.

#### Family Onchidiidae (Gastropoda)

In Hong Kong, Britton [65] recorded four species, namely *Peronia verruculata*, *Paraonchidium reevesii*, *Platevindex mortoni* and *Onchidium hongkongense*. As previously mentioned, a revision on the taxonomy of this family is being carried out by Dayrat et al. [44–46, 66–69], and found that *P. reevesii*, *O. hongkongense*, and some of the *P. verruculata* in [65] are actually *Paromoionchis tumidus* [44, 66]. Our molecular analyses revealed six species of Onchidiidae in Hong Kong (Table 1).

#### Family Potamididae (Gastropoda)

Recently, *Terebralia palustris* was reported in Hong Kong. However, no *T. palustris* was collected during our extensive surveys, as confirmed by both morphological and molecular analyses. Thus, this study shows that in Hong Kong mangroves the genus *Terebralia* is represented by *T. sulcata* only. On the bases of the taxonomic revision of the family Potamididae [21] and of the genus *Cerithidea* [70, 71] and of our molecular analyses we could only confirm the presence of *Cerithidea tonkiniana* and *C. moerchii* among the many species of the genus *Cerithidea* previous recorded from Hong Kong mangroves. It is worth noting that *C. sinensis* was also recorded in Hong Kong by Reid [70]. However, the species is mainly found in the Yellow Sea and it is not associated with mangroves [70]. Reid and co-workers also redefined the genus *Cerithideopsilla* [21], which was recently renamed and divided into 16 species of *Pirenella* (= *Cerithideopsilla*) from three clades [72, 73]. In Hong Kong, several species belonging to this genus were recorded, identified, misidentified and renamed in the recent years [28, 32, 61, 74], but our study could only confirm the presence of five species (see Table 1 and Additional file 1: Table S1). *P. microptera* was also recorded in Hong Kong by [72] but seems to be rare and not directly associated with mangrove forests.

#### Family Turbinidae (Gastropoda)

*Lunella coronata* was recorded in Hong Kong [28, 32] but here we confirm that it was a misidentification for *L. granulata*, which occurs on mainland Asia, Taiwan and Okinawa [75].

#### Family Sesarmidae (Brachyura)

*Parasesarma plicatum* and *Neosarmatium smithi* were thought to be present in Hong Kong mangroves, although their distribution ranges did not include the Southern China sea. Here we showed that the specimen recognised as *P. plicatum* and *N. smithi* in Hong Kong belong to the species *P. ungulatum* and *N. indicum*, respectively, confirming the information available in the literature [76, 77].

#### Family Xanthidae (Brachyura)

*Leptodius exaratus* has previously been recorded in Hong Kong [78]. The species was reviewed in [79] where the authors concluded that *L. exaratus* only occurs in the western Indian Ocean, while the populations from the eastern Indian Ocean and western Pacific Ocean are in fact *Leptodius affinis*, such as our specimen.

We found that some misidentifications and taxonomic inconsistencies reported in the present study also correspond to inaccurate barcoding data deposited in the GenBank or BOLD databases. Noteworthy, most of the comparisons of our crab sequences to those publicly available were accurate and led to reliable confirmations of our morphology-based species identifications. Conversely, the situation was more problematic for gastropods, for which we could unveil several misleading records. This issue can be ascribed to the fact that various families of mangrove associated gastropods are now undergoing extensive taxonomic and systematic revisions and, therefore, sequences uploaded in past years still refer to the old classifications. At the same time, we cannot ignore that GenBank is just a sequence repository and does operate a limited “quality check” on the uploaded sequences, resulting in many documented mistakes in terms of sequencing errors, pseudogenes and species identification (for a review see [80]). Despite these known issues, we recognise the utility of the BLAST search tool in the sequence databases, provided that careful control of taxonomic identification and an update of the species names in accordance with taxonomic revisions and rearrangements are carried out. Conversely, uncritical use of sequence repositories can lead to errors that are perpetuated over time.

### The three-gap deletion of Ellobiidae and Onchidiidae

Indels are usually very rare in the mtDNA COxI gene [81]. This rule does not seem to apply to molluscs for which various indels have been described in different lineages of Gastropoda and Bivalvia [82]. All the described indels are in multiples of three nucleotides and are primarily in the region of the first external loop of the protein. This suggests that in molluscs this part of the gene is particularly susceptible to insertions and deletions and that the gene has an accelerated mutation rate [82]. The three-gap deletion in position 106–108 bp that we detected in the sampled species of the family Ellobiidae and Onchidiidae has been already reported in Onchidiidae and Pyramidellidae (Gastropoda) as well as Tellinidae (Bivalvia) [82]. The detection of this deletion may serve for a rapid taxonomic assignment of mangrove gastropods at family level, being only present in two out of 13 families occurring in this geographic area.

### Further utility of the present barcode library

Traditionally, identification of marine larvae or juvenile stages of brachyuran crabs and molluscs, even to order level, is a complex and time-consuming task. Ontogenic changes across larval stages make the identification even more difficult and require high levels of expertise. Despite this, a correct identification of larvae is crucial when studying patterns and processes influencing marine populations, communities and ecosystems. This barcode library can help solving the above-described shortfalls for mangrove-associated molluscs and crabs thriving in the Greater Bay Area, since it makes available for comparison a set of COxI sequences from adults, morphologically and genetically assigned at species level. For mangrove crabs, this approach proved to be successful for East African species [83].

Our barcode library will also prove to be a critical tool for studying the Greater Bay Area mangrove biodiversity and monitoring future changes through environmental DNA (eDNA) sequencing approaches. eDNA analysis, based on Next Generation Sequencing (NGS) techniques, is a rapid and non-invasive approach to identify species present in a given environment. It can solve some limitations of traditional approaches for biodiversity monitoring, such as extensive field surveys and difficulties in morphological species identification [84]. The method relies on the fact that organisms shed DNA into their environments and thus genetic materials can be obtained directly from environmental samples such as soil, sediment and water. Due to the lack of barcode databases such as the present one, eDNA metabarcoding is still a new and developing approach for mangrove-associated fauna, and the few available studies mainly investigate mangrove fish diversity [85, 86].

## Conclusion

This study is the first attempt to provide a reference barcode library for mangrove-associated macrobenthic fauna of Hong Kong and the whole Greater Bay Area and represents a reliable tool to assist researchers and managers in their conservation efforts. Macrobenthos diversity is in fact a recognised indicator of the state, functionality, and health of mangrove forests [7]. Mangrove-associated macrobenthic fauna perform crucial ecosystem functions, such as their bio-engineering activities on the sediment that ultimately influence nutrient cycling and sequestration [6, 87]. The ongoing ecological degradation of mangrove forests, but also some consequent rehabilitation schemes that ignore best practices, pose a great risk to the specialised and rich fauna uniquely linked to these ecosystems, a risk that, in turn, can impair critical functions linked to the macrobenthos itself.

## Methods

### Sampling sites and ecological characterisation

Mangrove forests in Hong Kong has expanded from an estimated 178 hectares in 1997 [88] to more than 500 hectares in 2020 [10]. Apart from the extensive mangrove forest of the Mai Po Nature Reserve, the largest in the Greater Bay Area, the remainder consists of small mangrove strands of less than 10 hectares in extension, colonising the numerous small bays and inlets of Hong Kong’s coastline. The mangrove trees and invertebrates thriving on the western coast of Hong Kong face the Pearl River Estuary, characterised by low salinity and high levels of nutrients, especially during the wet season [89]. Comparatively, the eastern coast of Hong Kong is more oceanic and is characterised by higher salinities and lower nitrogen concentrations and hosts small patches of fringing mangroves.

### Sampling and morphological identification

**Fig. 1:**
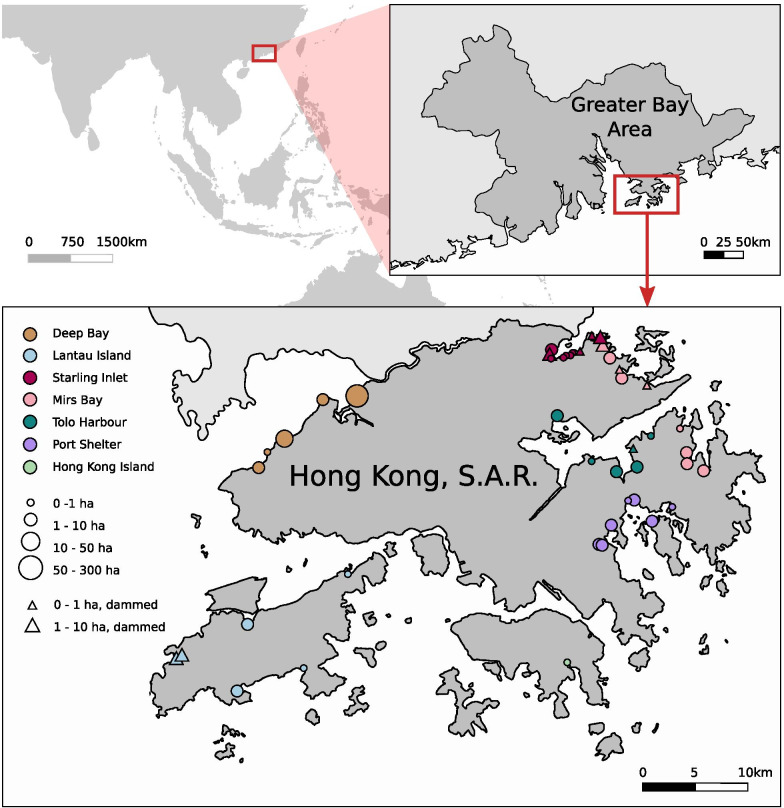
Maps showing the geographical location of the study area and detailed map of the sampling area showing the 44 mangrove patches surveyed for this study. Different colours of the circles refer to different geographic area, while symbol size is proportional to mangrove extension. Triangles indicate mangrove forests grown behind a dam

Sampling surveys for mangrove crabs and gastropods took place from 2017 to 2020 during the wet season, i.e., from June to September. Hong Kong has a subtropical climate with limited seasonal variations in temperature that do not affect the faunal composition of its coastal habitats [28], but mangrove crabs are known to be more active during the warm wet season [90]. A total of 44 Hong Kong mangrove patches along the West and the East coasts (Fig. 1) were visited multiple times, during low spring and neap tides and at different times of the day and night. Each survey was performed by at least two expert researchers and the time spent during each visit was proportional to the size of the site, varying from 30 min to 2 h, to account for the differences in area of the various sites. The sites were investigated from the landward to the seaward side, and all possible microhabitats (sediment, rocks, debris, tree trunk and branches, roots, foliage and litter) were inspected.

A total of approximately 1100 specimens (300 gastropods and 800 crabs) were collected and transported to the laboratory for identification and, in some cases, subsequent DNA extraction. Gastropods were removed from their shell and preserved in absolute ethanol, while for each crab either a cheliped or a pereiopod was removed and preserved in absolute ethanol before conserving the entire specimen in 75% ethanol. All specimens were identified to species level a priori on the basis of their morphological characteristics using the most updated taxonomic literature available [31, 33, 34, 42, 44–48, 51, 52, 60, 61, 64, 66, 68–73, 76, 77, 91–106]. Some specimens were transported to Lee Kong Chian Natural History Museum, National University of Singapore, to compare them with the type specimens preserved in the museum collections for an accurate identification. Voucher specimens were deposited in the Swire Institute of Marine Science (SWIMS) of the University of Hong Kong, the Zoological Reference Collection (ZRC) of the Lee Kong Chian Natural History Museum, National University of Singapore, Singapore, and the Natural History Museum of the University of Florence (MZUF), Florence, Italy (voucher numbers provided in Additional file 1: Table S1).

### Genetic analyses

After the a priori species identification, one or more specimens of each species, for a total of 285 (130 gastropods and 155 crabs) specimens, were selected for molecular analyses. Total genomic DNA was extracted from muscle tissue, removed from one pereiopod and from the foot for brachyuran crabs and gastropods, respectively, using the Qiagen QIAmp tissue kit. After precipitation, DNAs were re-suspended in sterile distilled water and stored at 4 °C for routine use, or at – 20 °C for long-term storage. Selective amplification of 658 basepairs of the mtDNA COxI was performed with the primers LCOI1490 (5′-ggtcaacaaatcataaagatattgg-3′) [107] or COL6b (5′-acaaatcataaagatatygg-3′) [108] in combination with the primer HCO2198 (5′-taaacttcagggtgaccaaaaaatca-3′) [107]. The amplifications were performed in a final volume of 20 μL using 2 mM of MgCl_2_, 300 μM of dNTPs, 0.6 μM of each primer and 0.4 U of Taq polymerase in a Perkin Elmer 9600 thermal cycler. Thermal profiles consisted of an initial denaturation step at 94 °C for 5 min, followed by 35–40 cycles of denaturation for 45 s at 94 °C, annealing for 45 s at 47–49 °C and extension for 45 s at 72 °C, with a final extension step of 10 min at 72 °C. Then, amplicons were visualised on an 1% agarose gel, purified by precipitation with Sure Clean (Bioline) and re-suspended in water. The sequence reactions were performed with the ABI BigDye terminator mix. Then sequencing products were isopropanol-precipitated and resolved by capillary electrophoresis in an Applied Biosystems 3130xl genetic analyser.

The COxI sequences were manually corrected and aligned using Geneious 11.0 [109]. Each sequence was then compared to sequences deposited in the NCBI database (National Center for Biotechnology Information) through the Geneious command *blastn*. We also compared the new sequences to our own previous reference sequences. All original haplotypes were submitted to molecular databases (accession numbers provided in Additional file 1: Table S1). The crab’s dataset was integrated with three sequences retrieved from GenBank. A sequence of *Sinosesarma tangi* (Sesarmidae), was obtained from a specimen collected during the present survey, published in a previous paper [93] and deposited in GenBank with the accession number LC500793. Its museum voucher is deposited at the Zoological Collections of the National Chung Hsing University (NCHUZOOL), Taichung, Taiwan. The other two sequences (LC097123 and LC097124) corresponded to *Macrophthalmus erato* (Macrophthalmidae), the only crab species for which we did not retrieve reliable barcode sequences from our samples. Morphological assignment of these two sequences is definitely reliable as it was made by expert colleagues in crab taxonomy (Prof. Shih H.T. and Teng S.J of the National Chung Hsing University, Taiwan).

### Molecular distance analysis and MOTUs delineation

**Fig. 2:**
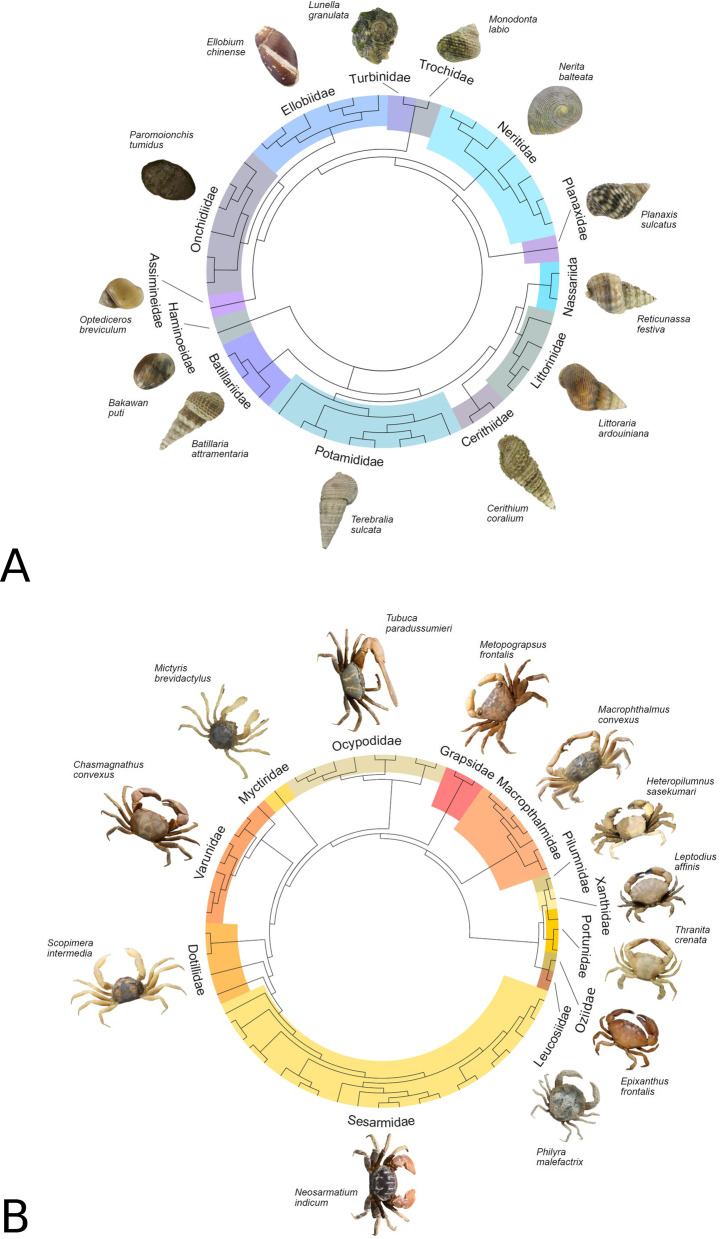
Representation of the mollusc (**A**) and brachyuran crab (**B**) taxa covered in the study. Cladograms were produced using K2P distances and according to the most up-to-date information on crab and mollusc taxonomy [20, 21, 45, 113]. Photographs by S. Cannicci and C. L. Y. Cheng

A number of statistical analyses were conducted in parallel on gastropod and crab datasets to assess molecular distances and delineate Molecular Operational Taxonomic Units (MOTUs). Genetic distances between corrected haplotypes were estimated with MEGA7 [110], using a Neighbor-Joining [111] algorithm with the Kimura-2 parameter model (K2P) [112]. The robustness of nodes was evaluated through bootstrap re-analysis of 1000 pseudo-replicates. K2P distances were used to generate cladograms to provide a graphic representation of the taxa covered in the study (Fig. 2). Cladograms follow the most up-to-date information on brachyuran crab and gastropod systematics [20, 21, 45, 113].

Barcode sequences were also used to delineate MOTUs. Three species-delimitation methods were employed: the automatic barcode gap discovery (ABGD) method [114], the general mixed Yule coalescent (GMYC) model [115] and a Bayesian implementation of the Poisson tree processes (bPTP) model [116]. The three methods work without any a priori knowledge of species identity and were developed to approximate putative species. The ABGD method partitions sequences into groups based on comparisons of pairwise distances. We performed ABGD analysis using the web interface (https://bioinfo.mnhn.fr/abi/public/abgd/) with default settings (prior maximal intraspecific distances between 0.001 and 0.1) and using uncorrected p‐distances to automatically detect gaps in the distribution of pairwise distances among DNA barcodes.

The GMYC (single threshold approach) and the bPTP models are based on the phylogenetic species concept. The first identifies the transition points between inter- and intra-species branching rates on a time-calibrated ultrametric tree, while the second is based on a transition in the number of substitutions and does not require an ultrametric tree.

The phylogenetic trees used for GMYC and bPTP were built using Bayesian Inference (BI) of phylogeny and the Maximum Likelihood (ML) method, respectively. BI analysis was conducted with BEAST2 v2.6.3 [117] using the Yule model and a constant clock. The ML analysis was conducted using RAxML v8.2.10 [118] under the GTRGAMMAI evolutionary model with 1000 bootstrap pseudo-replicates.

## Supplementary Information

**Additional file 1.** Taxonomic characterization, GenBank accession numbers, museum vouchers ID and details of the sampling sites of all the specimens sequenced for this study.

**Additional file 2.** Sequence alignment of the brachyuran crab barcode COxI regions.

**Additional file 3.** Sequence alignment of the mollusc barcode COxI regions.

## Data Availability

The DNA sequence dataset supporting the conclusions of this article is available in the NCBI database (accession numbers provided in Additional file 1). The sequence alignment of the barcode COxI regions of crabs and molluscs analysed in this study are reported in Additional files 2 and 3, respectively.

## Abbreviations

ABGD: Automatic barcode gap discovery
BI: Bayesian Inference
BOLD: Barcode of Life Data System
bPTP: Bayesian implementation of the Poisson tree processes
COxI: Cytochrome c oxidase subunit 1
DNA: Deoxyribonucleic acid
dNTPs: Deoxynucleotide triphosphates
eDNA: Environmental DNA
GMYC: General mixed Yule coalescent model
K2P: Kimura-2 parameter model
ML: Maximum Likelihood
MOTU: Molecular Operational Taxonomic Unit
MtDNA: Mitochondrial DNA
MZUF: Natural History Museum of the University of Florence
NCBI: National Center for Biotechnology Information
NCHUZOOL: Zoological Collections of the National Chung Hsing University
NGS: Next Generation Sequencing
PCR: Polymerase Chain Reaction
SWIMS: Swire Institute of Marine Science
ZRC: Zoological Reference Collection
